# Health service organisation impact on lower extremity amputations in people with type 2 diabetes with foot ulcers: systematic review and meta-analysis

**DOI:** 10.1007/s00592-020-01662-x

**Published:** 2021-02-06

**Authors:** Bernardo Meza-Torres, Fabrizio Carinci, Christian Heiss, Mark Joy, Simon de Lusignan

**Affiliations:** 1 Department of Clinical and Experimental Medicine, University of Surrey, Guildford, UK; 2 Nuffield Department of Primary Care Health Sciences, University of Oxford, Oxford, UK; 3 Department of Statistical Sciences, University of Bologna, Bologna, Italy; 4 Surrey and Sussex Healthcare NHS Trust, East Surrey Hospital, Redhill, UK

**Keywords:** Type 2 diabetes, Diabetic foot ulcers, Health service organization, Lower extremity amputation

## Abstract

**Aims:**

Despite the evidence available on the epidemiology of diabetic foot ulcers and associated complications, it is not clear how specific organizational aspects of health care systems can positively affect their clinical trajectory. We aim to evaluate the impact of organizational aspects of care on lower extremity amputation rates among people with type 2 diabetes affected by foot ulcers.

**Methods:**

We conducted a systematic review of the scientific literature published between 1999 and 2019, using the following key terms as search criteria: people with type 2 diabetes, diagnosed with diabetic foot ulcer, treated with specific processes and care pathways, and LEA as primary outcome. Overall results were reported as pooled odds ratios and 95% confidence intervals obtained using fixed and random effects models.

**Results:**

A total of 57 studies were found eligible, highlighting the following arrangements: dedicated teams, care pathways and protocols, multidisciplinary teams, and combined interventions. Among them, seven studies qualified for a meta-analysis. According to the random effects model, interventions including any of the four arrangements were associated with a 29% reduced risk of any type of lower extremity amputation (OR = 0.71; 95% CI 0.52–0.96). The effect was larger when focusing on major LEAs alone, leading to a 48% risk reduction (OR = 0.52; 95% CI 0.30–0.91).

**Conclusions:**

Specific organizational arrangements including multidisciplinary teams and care pathways can prevent half of the amputations in people with diabetes and foot ulcers. Further studies using standardized criteria are needed to investigate the cost-effectiveness to facilitate wider implementation of improved organizational arrangements. Similarly, research should identify specific roadblocks to translating evidence into action. These may be structures and processes at the health system level, e.g. availability of professionals with the right skillset, reimbursement mechanisms, and clear organizational intervention implementation guidelines.

**Supplementary Information:**

The online version contains supplementary material available at 10.1007/s00592-020-01662-x.

## Introduction

Lower extremity amputations (LEA) represent one of the most challenging complications experienced by people with type 2 diabetes (T2D). However, recent studies show a reduction of major amputations, this has been attributed to improved quality of health care provided over the last 15 years. Still, this contrasts with minor amputations, which in recent years have shown rates with stagnant declines or even slight increases, broadening the scope to improve outcomes [1–3].

Diabetes-related foot ulcers (DFU) are associated with higher rates of complications, in particular, renal failure, LEA, and disability. It has been shown that only between 50 and 60% of people affected by DFU survive more than 5 years after their first diagnosis [4, 5]. The cost of DFUs is estimated as one-third of the total costs of diabetes-related treatment [4, 6].

Despite the evidence available on the epidemiology of foot ulcers and associated complications [7–9], it is not clear how specific organizational aspects of health care systems can positively affect the trajectory between the first diagnosis of DFU and LEA.

In this study, we aimed to conduct a systematic review investigating the effectiveness of processes of care following the initial presentation of DFUs, as measured by the change in the rate of LEA over time.

This paper aims to respond to the following research questions:

- Which organizational factors are associated with reduced LEA rates following initial diagnosis of DFU in patients with type 2 diabetes?
- Which of these organizational arrangements can be pooled in a meta-analysis?

## Materials and methods

The population in included studies were adults diagnosed with T2D who presented with a foot ulcer as an index event. The occurrence of any lower extremity amputation was the primary endpoint. Major amputations were considered separately as a clinically relevant endpoint.

We conducted a systematic search on July 2019 using PubMed Medline and Excerpta Medica database (EMBASE), targeting studies published between 1999 and 2019. The search comprised four, main semantic components: (1) patients with diabetes, (2) with diabetic foot ulcer, (3) in the context of processes and care pathways, and (4) experiencing LEA or ulcer healing as potential negative/positive outcomes.

The search strings used in each database have been combined as follows: “Diabetes Mellitus” [MeSH] AND ("Diabetic Foot"[Mesh] OR (diabet*[tiab]) OR "Foot Ulcer"[Mesh] OR (foot ulcer*[tiab]) OR Leg Ulcer [MeSH]) AND ((process*OR pathway*) AND care) AND ("Amputation"[Mesh] OR (amputation*[tiab]) OR (Lower extremity amputation*[tiab]) OR (LEA[tiab])).

To be eligible, each study had to include an organizational arrangement as a specific intervention of interest, considered as the main exposure.

To help interpretation, we classified the identified organizational arrangements according to the Donabedian model for evaluating quality of care [10]. This framework for health systems research indicates how modifying the structures and processes at the healthcare system or provider level can predict patient-level outcomes. Thus, this study considered prevention strategies implemented at the care provider level. Therefore, patient-level interventions alone were not considered, e.g. standalone patient education strategy.

Our classification was drawn from the approach adopted by recognized institutions, including the TRIAD Study Group [11, 12], the UK’s National Institute for Health and Care Excellence [13–15], the International Consortium for Health Outcomes Measurement [16], the International Working Group on the Diabetic Foot [17], and various authors’ view on integrated care [5, 18].

We created the following categories of organizational arrangements:

- Dedicated teams: involving the implementation of human and structural resources, e.g. specialty nurses and dedicated inpatient beds, to specifically treat diabetic foot ulcers.
- Multidisciplinary teams: involving an integrated framework of multispecialty care, whose resources might not be exclusive to DFU. They are coordinated to manage DFU through a care plan, a key worker/manager, and joint activities, e.g. foot clinics integrating diabetology, podiatry, specialty nurses, vascular/orthopaedic surgery, microbiology, wound care, nutrition, etc.
- Care pathways: including explicit and dedicated step-by-step protocols for the local care team to treat a patient in a specific setting, e.g. risk profiling, access to prevention and treatment, referencing systems, and a clinical setting for chronic disease monitoring.
- Combined interventions: complex interventions integrating the above elements without a predominant one. Since only arrangements implemented at the level of care providers were considered, the presence or absence of patient-level interventions implemented alongside was not considered for this categorization, e.g. patient education interventions.

The literature review was conducted according to the guidelines of the Preferred Reporting Items for Systematic Reviews and Meta-Analyses (PRISMA) [19] Flow and the Cochrane Handbook for Systematic Reviews of Interventions v.5.1. [20].

We considered as eligible study designs all controlled or observational studies, either prospective or retrospective, as well as systematic reviews or meta-analyses. Narrative reviews, clinical practice guidelines, case series, case reports, or letters to editors were excluded from the search.

### Classification of eligible studies

The full text of the selected papers was retrieved and assessed for eligibility by co-authors BMT and FC independently. Study eligibility for qualitative review was based on the following characteristics: (a) participants (people with T2D presenting with a foot ulcer as an index condition); (b) intervention (specific organizational arrangements); and (c) outcome (LEA as primary endpoint). The percentage of agreement was assessed using Cohen’s kappa statistics [20].

Once selected, studies were stratified by BMT according to the type of quantitative outcome reported (e.g. binary outcome, OR, and RR), and by the category of organizational arrangements (e.g. dedicated teams, care pathways, multidisciplinary teams, and combined interventions). The list of references included in the identified studies was also examined for the inclusion of additional studies. Their eligibility for binary outcome meta-analysis was based on the following criteria: (a) studies presenting results in terms of quantitative measures; (b) quantitative measures must report the number of LEA cases as well as the number of persons at risk (population as denominator, not as rate); (c) and the quantitative measures should be reported for both an intervention and a control group (either in parallel or before vs after). The data collection form is included as supplement 1.

The risk of bias assessment was conducted on all studies finally selected, aided by the Newcastle–Ottawa Scale for cohort and case–control studies and the Cochrane Collaboration’s tool for those studies with the respective designs [21, 22]. Publication bias was assessed through funnel plot asymmetry analysis [20].

Quantitative assessment was carried out using a fixed and random effects meta-analytical approach for studies presenting results in terms of quantitative measures, e.g. the number of cases and number of people at risk separately, for both intervention and control groups. Overall results were calculated using a Mantel Hansel approach, weighted by the inverse variance [20]. We based our presentation of key results via fixed or random effects models, based upon the significance of the Q test of heterogeneity.

All statistical analyses were performed using the statistical package R v.1.2 and the *metafor* package [23, 24].

## Results

**Fig. 1:**
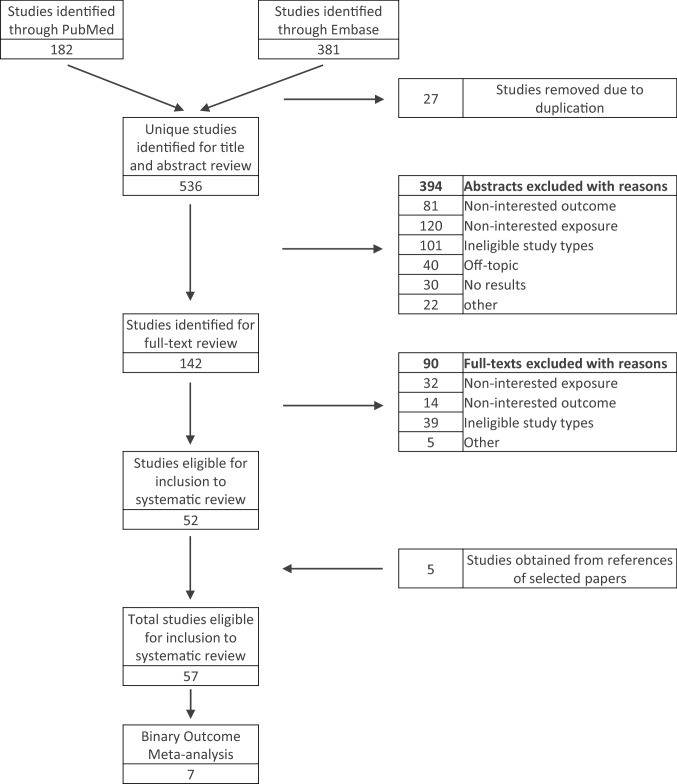
Diagram of selection of eligible studies

As shown in Fig. 1, a total of *N* = 536 unique studies were initially identified from Medline and Embase, based on the above-specified search strategy. Following further examination, a total of 142 articles were retrieved for full-text review, of which further 90 were excluded due to unrelated exposure (*N* = 32), unrelated outcome (*N* = 14), ineligible study types (*N* = 39), and other reasons (*N* = 5). Additionally, five studies were added by cross-referencing the retrieved studies. At the end of the process, a final subset of 57 articles were selected for review in the present article. The percentage of agreement between authors was of 56%. The different organizational factors investigated in the final set of papers, along with the type of evidence supporting each of them, are shown in cross-tabulation in Table 1. The individual studies were graded as ‘strong’ when their findings were reported quantitatively, and as ‘weak’ when only narrative or non-significant quantitative results were reported. Due to heterogeneity in study designs, a single quality assessment tool could not be used to compare amongst the 57 studies. Hence an independent assessment by two authors was used.

**Table 1:** Classification of results obtained from the literature review

Evidence	Care pathways	Combined intervention	Dedicated teams	Multidisciplinary teams	Total
1. Strong against	**-**	**1** (5%)	**–**	**–**	**1** (2%)
2. Weak against	**2** (10%)	**1** (5%)	**–**	**–**	**3** (5%)
3. Inconclusive evidence	**-**	**5** (24%)	**–**	**–**	**5** (9%)
4. Weak support	**9** (45%)	**10** (48%)	**1** (100%)	**6** (40%)	**26** (46%)
5. Strong support	**9** (45%)	**4** (19%)		**9** (60%)	**22** (39%)
**Total**	**20** (100%)	**21** (100%)	**1** (100%)	**15** (100%)	**57** (100%)

*N* (percentage)

‘Weak’: supporting evidence with low statistical significance, or narrative support

‘Strong’: supporting evidence with statistical significance

The studies investigated the following organizational factors: care pathways, dedicated teams, multidisciplinary teams, and combined interventions.

Among the 57 studies identified, a total of *N* = 41 (72%) reported an intervention that could be either categorized as a care pathway or combined intervention (combining care pathways with other interventions at the care provider level). Most combined interventions included care pathways coupled to multidisciplinary teams where specialists—in primary or secondary care—were integrated with a multidisciplinary setting. In some cases, combined interventions also included educational strategies in the outpatient setting.

Twenty of the studies (35%) focused on interventions classified as care pathways, characterized by a protocol including multiple specialists that were not articulated within the same setting. Among them, *N* = 9 (16%) reported strong evidence of improvement. The remaining 11 studies (19%) either showed weak support, including narrative argumentation and non-significant results, or in just two papers no evidence of an effect of care pathways on the reduction of LEA. A justification given for the lack of effect was that pathways may be complex to implement as they traverse organizational structures, creating barriers in the patient’s journey.

Twenty-one papers (37%) focused on the effect of combined interventions. Among them, four papers reported a statistically significant reduction in LEA rates, while ten addressed combined interventions including approaches of patient-centred care. In this subgroup of papers, one study found a lack of response of octogenarians with PAD to total amputation rates, independently from improvements in care and education.

A total of *N* = 15 studies (18%) reported the effect of multidisciplinary teams, all of them showing improvements. In some cases, it was shown that the multidisciplinary approach may be associated with increased workload, making the case for further cost-effectiveness research. However, these studies showed a remarkable heterogeneity in the composition of their teams, many involving primary and secondary care specialists (podiatry, nurses, vascular surgeons, and diabetologists) who were coordinated in the same physical space on certain days.

Dedicated teams were found only in one study, showing no statistical significance, but suggesting that podiatry services can help reducing LEA rates in selected high-risk subgroups.

**Fig. 2:**
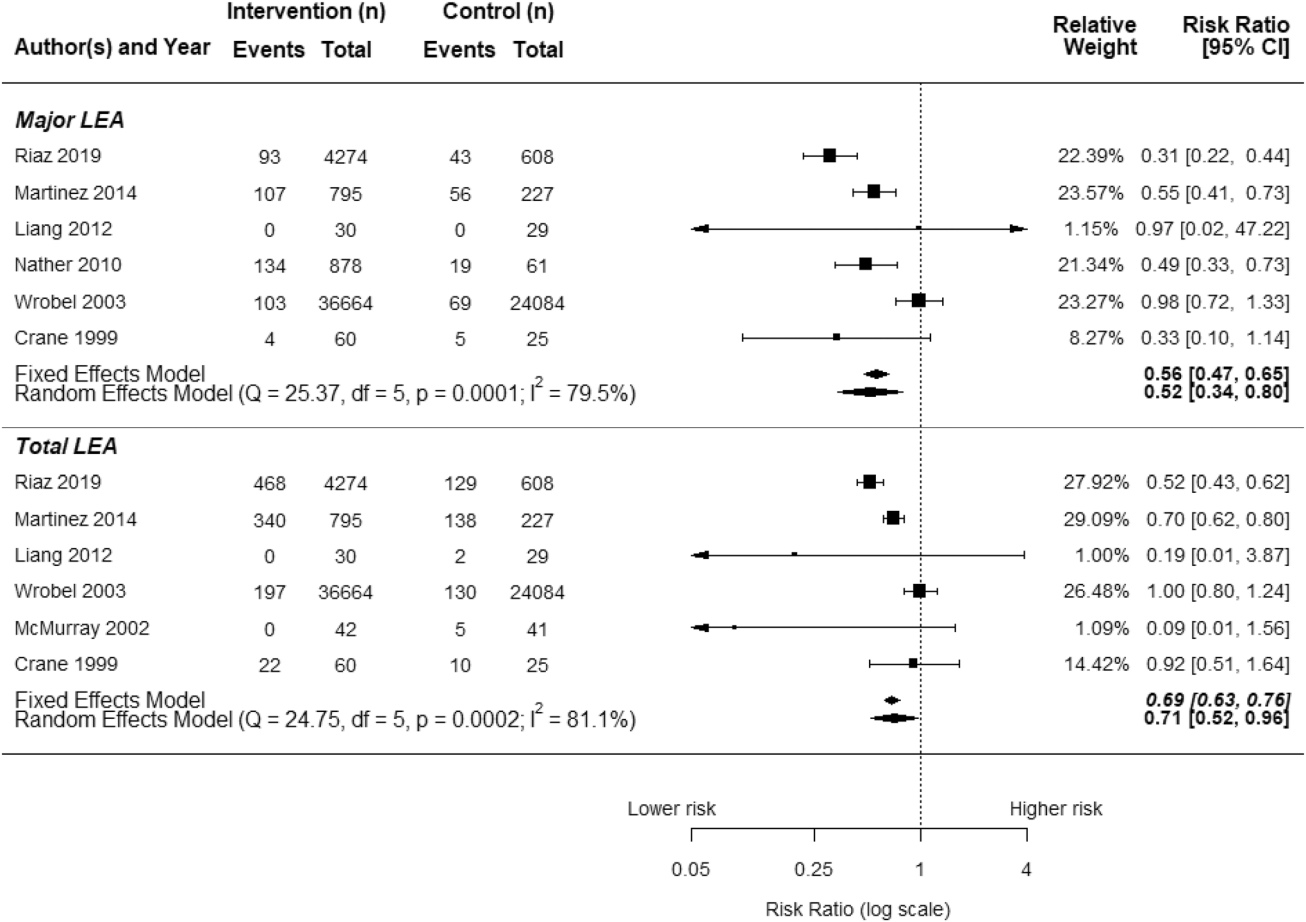
Forest plot of quantitative meta-analysis of eligible studies

Only seven of the studies were included in the meta-analysis [25–31], as they reported the number of cases of LEA as well as the population denominator (not as rate) for both the intervention and control groups (either in parallel or before vs after). The results obtained from the meta-analysis are presented in Fig. 2. The majority of these studies investigated combined interventions (*N* = 4), while the others focused on care pathways (*N* = 2) and multidisciplinary teams (see Table 2). Given the limited number of studies, we could not report results by type of organizational arrangement, but only in terms of organizational intervention yes/no.

**Table 2:** Description of meta-analyzed studies

Study acronym	Crane 1999	Liang 2012	Martinez 2014	McMurray 2002	Nather 2010	Riaz 2019	Wrobel 2003
Intervention	Care pathways	Multidisciplinary teams	Combined intervention	Combined intervention	Combined intervention	Combined intervention	Care pathways
Duration of the study	3 years (1993–1996)	2 years (2006–2008)	14 years (1998–2012)	1 year (date not specified)	5 years (2002–2007)	19 years (1997–2016)	1 year (2000–2001)
Comparison	Before versus after intervention	Intervention versus usual care	Before versus after two interventions	Intervention versus usual care	Before versus after multicomparison	Before versus after intervention	Best versus worst performers in foot care effectiveness score
Reporting of outcomes	Minor, major LEA	Total LEA	Minor, major, total, elective, urgent LEA	Major, total LEA	Major, total LEA	Minor and major (by anatomic region), total LEA	Minor, major, total LEA
Case-mix variables	Age, sex	Age, sex, literacy, ethnicity. Mainly urban areas	Age, sex	Age, sex	Age, sex	Age, sex	No information provided
Patients’ risk level and severity of the clinical presentation	ICD-9: diabetics admitted with foot complications	ADA 1998: includes risk 1–3 patients	ICD-9: diabetics with foot complications; LEA	Unspecified foot risk classification	King's College classification 3–5, diagnostic related group	DFU diagnosis; diabetics with DFU attending foot clinic	ICD-9: identify LEA patients
Setting of care	Hospital, inpatient	Hospital, inpatient. The study argues that this is a primary care setting, since in China primary care is often provided at an inpatient setting	Hospital, ER	Dialysis unit	Inpatient hospital clinic	Tertiary care unit, hospital	VA hospital clinics, primary care
Stage of managed care	Improving the healing, secondary prevention—approach to emergency room patients	Primary prevention	Improving the healing, secondary prevention	Improving the healing, secondary prevention	Improving the healing (inpatient treatment), secondary prevention (discharge plan)	Improving the healing, secondary prevention	Improving the healing, secondary prevention
Study design	Retrospective	Randomized clinical trial	Retrospective incidence study	Prospective interventional	Retrospective	Retrospective	Cross-sectional survey
Results (minor vs major LEA)	Significant decrease in the proportion of major amputations (BKA or AKA) in 1995 and 1996 as compared to baseline values (1993 = 23%, 1995–1996 = 7%, *p* = .02) Significant decrease in the proportion of major amputations 1995–1996 (pathway = 7%, non-pathway = 29%, *p* < .00 l) There was not a significant difference in minor amputations (non-significant increase)	Non-significant difference for total LEA (5 amputations in control group vs 0 in intervention group). Minor LEA not reported	Overall decrease in total and major LEA. Very significant decrease in elective major LEA. Between the first period and last period total LEA fall 32.8% (*p* = 0.003). Major LEA fall 46.4% (*p* < 0.001), electives major LEA fall 57.7% (*p* < 0.001) and minor LEA fall 13.3% (*p* = 0.199) No significant difference for minor. group B versus C + 6.4 increase in minor *p* = 0.5	Non-significant decrease in minor and major amputations Significant decrease in risk progression	Significant decrease in major amputations, total not reported. 13.11 to 8.26% *p* = 0.0009	When comparing before vs after intervention there is a significant decrease in toe (from 13.81% to 8.11% p0.042), below knee (from 5.26% to 1.82% p0.03), above knee amputations (from 1.8% to 0.35% p0.008) Significant increase in transmetatarsal amputations (from 0.3% to 0.65% *p* = 0.041)	Non-significant difference in minor LEA
Age (mean)	Control: 70.2(42–95) Intervention: 63.7(32–93)	Control: 55.8(20–68) Intervention: 56.2(22–70)	Control: 65 + 17 Intervention: 67 + 17 Median age 67 + 17	Control: 60.9 + 11.7 (control) Intervention: 63.0 + 13.5 p0.293	Both groups: 53.8 + -10.4	Control: 52.7 + 10.3 A(control, before) Intervention: 53.84 + 10.47 B	No age and gender reported
Gender (% male)	Control: 46% Intervention: 61%	Control: 19/29 Intervention: 14/30	Control: 58% Intervention: 66%; *p* = 0.002	Control: 55% Intervention: 53%; *p* = 0.86	Both groups: 70.7%	Control: 65.7% Intervention: 71.4%	No gender reported
Study population	Diabetic patients with chronic foot ulcer or gangrene as primary diagnosis (hospital records, ICD-10)	Diabetic patients with high ADA risk	Patients with diabetic foot complications admitted to hospital. Healthcare districts Murcia (170–240 inhabitants; 15-22 k diabetics), ICD-9	Diabetic patients undergoing dialysis	Patients with diabetic foot problems as according to King's College classification	Patients with DFU diagnosis	People with diabetes diagnosis from hospital discharge records
Intervention characteristics	Inpatient critical pathways: voluntary podiatry-only logarithmic approach to emergency room patients admitted with diabetic pedal infections	A diabetes nurse led multidisciplinary team was established, which included three endocrinologists, four nurses and one dietitian. In China, many diabetes complications are treated on inpatient basis	Complex pathway, multiteam, dedicated specialists, pathway (communication and referral system with primary care), treatment guidelines	Diabetes education programme and were followed up by a care manager who provided self-management education, diabetes self-care monitoring/management, motivational coaching, and foot checks	A diabetic foot team led by an orthopaedic surgeon and members of the team include an endocrinologist; an infectious disease specialist; a vascular surgeon; podiatrists; nurses specialised in wound care, foot care, and foot screening; and a case manage. multidisciplinary teams, implemented clinical pathway, weekly team ward round, and discharge plan	Implementation of training, staff, and pathway: footwear technicians, df assistants, standard operating procedure developed, physicians trained in diabetic foot surgery, patient education	Foot systems assessment tool: Level of programming and feedback of professionals within a sample of clinics in the USA
Quality appraisal	NOS = 8	Cochrane tool: low for selection and performance bias; high for other fields Other biases: limited follow-up period Small sample size and low representative of cases	NOS = 8	NOS = 7 Limited follow-up period	NOS = 8	NOS = 8	NOS for case–control studies = 7

Data extraction from hospital management system /hospital records

*DRG* diagnostic related group, *KCC* King’s college classification, *FCES* foot care effectiveness score, *NOS* Newcastle–Ottawa Scale for cohort studies

Overall, using the fixed model, we found that interventions including the four types of organizational arrangements were associated with a 31% reduced risk of any type of LEAs (OR = 0.69; 95% CI 0.63–0.76). Focusing on major LEAs alone, the result was equal to a 44% risk reduction (OR = 0.56; 0.47–0.65).

However, the result of the Q heterogeneity test was highly significant in both cases (*p* < 0.001).

Therefore, we applied the random effects model as a more conservative estimate, showing a 29% risk reduction of any type of LEAs (OR = 0.71; 95% CI 0.52–0.96) and a larger effect on major LEAs alone, equal to a 48% risk reduction (OR = 0.52; 95% CI 0.30–0.91).

Four studies [25–28] reported a decrease in the number of major LEAs while at the same time reporting either no difference or a slight increase in minor LEAs. Since data in these studies were aggregated, it was not possible to discern whether the increase in minor LEAs was due to multiple sequential minor amputations on the same patients.

Two of the studies with smaller sample sizes [30, 31] did not show statistically significant results (Fig. 2). Their small sample size is reflected in their lower quality scores (Table 2).

**Fig. 3:**
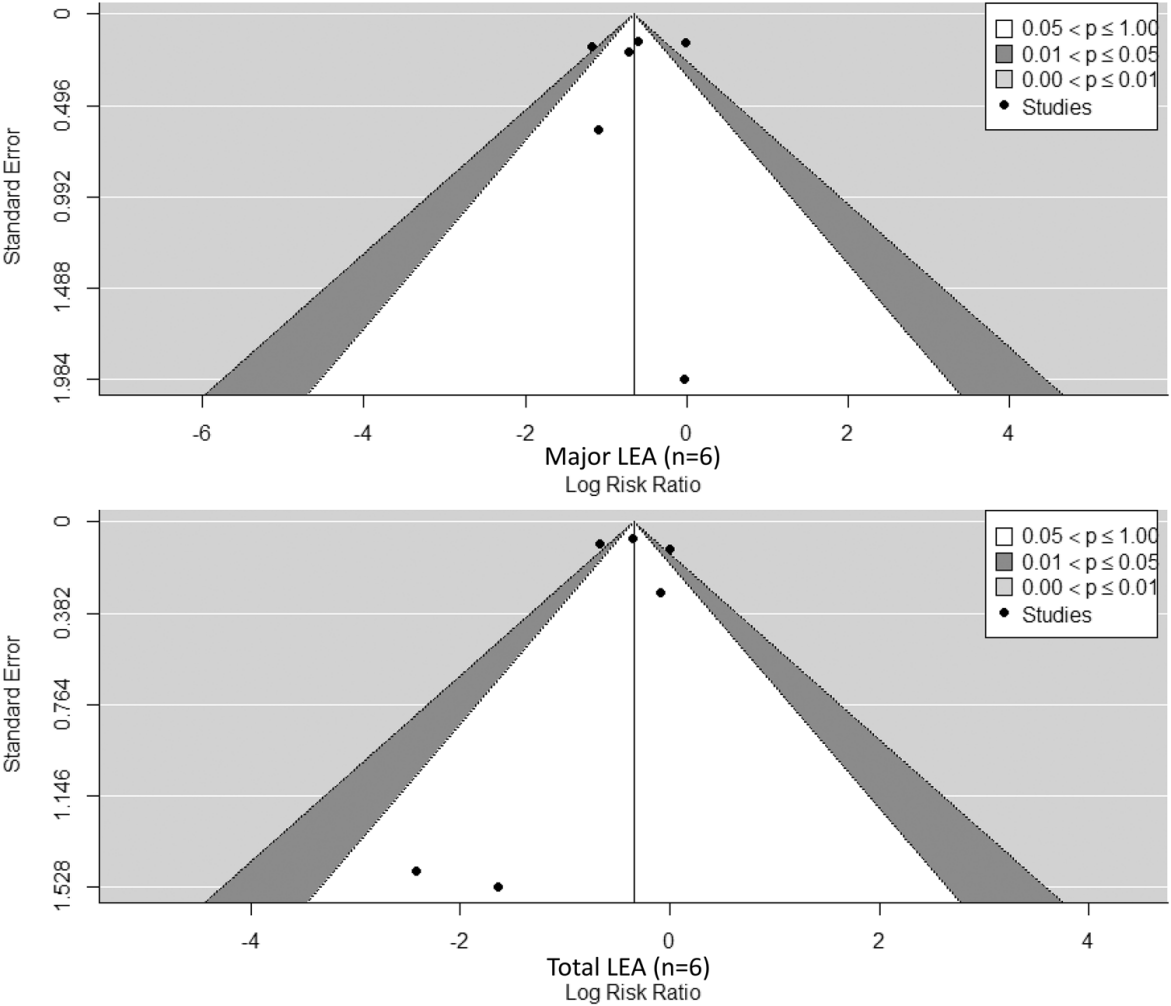
Publication bias funnel plots

The above observation is consistent with publication bias funnel plots (Fig. 3), which show small-study asymmetry on left lower quadrant for the Total LEA subgroup, accounted by these studies. Regression tests for asymmetry analysis were not significant (*p* > 0.2) which is expected due to the low number of studies (*n* < 10) [20].

## Discussion

Our systematic literature review identified fifty-seven eligible studies that have investigated the efficacy of different organizational factors in the management of people with diabetes and ulcers, namely care pathways, dedicated or multidisciplinary teams, and combined interventions. Overall, the majority of studies supported that these organizational factors can improve the outcome of patients with diabetes and ulcers.

### Main findings and generalizability

Despite evidence-based guidelines for the prevention and treatment of diabetes and foot ulcers [32, 33], amputations and mortality are still high. Studies suggest that this is in part because guidelines for recommended management (e.g. multidisciplinary care) are not implemented and also low adherence of patient to treatments. Integrated care pathways are intended to tackle these problems by breaking guidelines down into simple steps and assigning clear responsibilities to different specialties that should be involved. For instance, foot care is an integral part of the NICE diabetes pathway [13] and includes essential elements aimed at reducing the risk of developing and managing a diabetic foot problem. The majority of studies we identified showed that care pathways had a positive effect. Despite their heterogeneity, pathways shared an explicit and simpler depiction of the referral and treatment steps that are feasible under the already existing macro-level structures and processes of care (e.g. reimbursement mechanisms, practice guidelines), or by implementing feasible changes at the local level structures-processes of care.

People with diabetes and ulcers generally have multimorbidities associated with the vascular complications of diabetes. Therefore, effective treatment is diverse, requiring expertise across several medical and surgical specialties. Guidelines require that the effective management of ulcers includes a multidisciplinary team [32–34]. Our current analysis supports this. The team compositions were heterogeneous (see Table 2) and it remains to be determined if this approach is cost-effective and which team members or training requirements are essential. Vascular medicine has been proposed as an effective building block of such a team [35]. This, however, can only be further investigated on a background of comparable care pathways.

Our search also showed that combined interventions may be effective. While care pathways were a component of all combined studies, several included multidisciplinary teams and patient-based interventions. Since there may exist a definition overlap with different arrangements of integrated care, combined interventions are here useful to depict the natural development of either intervention type into an increasingly complex one, in terms of categories, breadth, and degree[18, 36–38]. But this complexity may also make combined interventions harder to implement as a first step in clinical settings. Similarly, their evaluation may face multiple confounding factors.

Finally, the scarcity of studies on dedicated teams (only one) can be explained by the fact that healthcare professionals may not exclusively be dedicated to attending DFUs but looking also after other conditions.

Regarding our second research question, we found that among the fifty-seven studies identified, only seven could match the criteria for inclusion in a quantitative meta-analysis.

These studies demonstrated that specific organizational arrangements were significantly associated with a reduction of major LEAs. However, our current meta-analysis showed that organized care can prevent almost half the major amputations. Previous cost-effectiveness studies show that cost-intensive interventions that prevent 50% of amputations result in net savings of $3000–4000 USD per patient [39]. The cost-saving impact could therefore be further investigated, considering that countries like England spend 0.9% of the National Health Service budget on the management of DFU and LEAs [40].

There was a different mix of study designs, namely: retrospective observational, prospective observational, and cross-sectional studies. Among them, observational studies had a longer duration and larger sample sizes. Therefore, their likelihood of reporting significant results was higher, with a larger magnitude of the effect that could be affected by bias [26, 27, 29]. Also, some observational designs reflect real-life data derived from electronic medical records (EMRs). This is important considering that experimental designs are hampered by the low incidence of LEA and long follow-up periods. There was also a mix in the study setting and stage of managed care. Observational studies more frequently included secondary care centres, e.g. hospital, emergency departments, and were more focused on secondary prevention, e.g. control of reulceration in patients already being treated for DFU. This is supported by the literature, where it has been argued that secondary prevention is plausible to be investigated because of the high 12-month reulceration rates (40%) among selected populations, such as those presenting with DFU complications requiring specialist care [5].

Among case-mix variables, age and sex were consistently reported in all except one study (Wrobel 2003). They homogenously report an average age > 50 years and male predominance. Other relevant demographic variables for case-mix adjustment, e.g. race or deprivation, were unreported, limiting population heterogeneity assessment and comparisons between studies.

In terms of DFU severity at baseline, only two studies reported a risk classification, e.g. King College Classification IV [29] and ADA 1998 [30]. All other studies only reported that patients had an established DFU diagnosis based on clinical codes. Ulcer grading is known to be linked to the outcome [5, 41] and should be considered for risk adjustment.

### Clinical relevance

Four studies described the use of care pathways specifically introduced for the study intervention, while three reported to follow just care protocols recommended by general guidelines. Multidisciplinary teams in each study also evaluated a unique combination of professionals, varying in the content, duration, and intensity of the intervention. Nonetheless, common elements across all studies included the presence of podiatrists, specialized nurses, and glucose control performed by endocrinologists or primary care physicians. Relevant heterogeneity included a variable involvement of surgical specialists, e.g. vascular or orthopaedic surgeons, and a different complexity of care pathways, e.g. communication and referral systems, discharge plans, and dedicated attention days.

Studies also presented variation in target outcomes. Four studies [25–27, 29] reported significant reduction of major amputations, associated with either an increase or a non-significant decrease in minor amputations. Due to the limitations of study reporting, it was not possible to assess the impact of patient profiles, e.g. multiple minor amputations performed on the same patient.

Actionability of the results emerged from this study should also be seen in the perspective of the future organizational evolution of diabetes care after the emergency of the COVID-19 pandemic, which took place well after this study was conceived and conducted. The disruption of care delivery may translate into an increase in LEA rates [42]. Furthermore, evidence is emerging that people with T2D are at a higher risk of serious complications due to COVID-19 [43], potentially presenting an opportunity to regard coordinated remote consultations for preventing infections in the household and primary care centers, while enhancing opportunities for multidisciplinary consultation.

### Relevance for future studies

The availability of structured healthcare databases using EMRs for surveillance [44] and quality of care monitoring [45] enables the construction of longitudinal cohorts on which clinical prediction models can be accurately validated. This approach can substantially reduce the cost of ad-hoc studies, while adjusting for relevant case-mix characteristics applying the Donabedian model considering both the individual and organizational levels. Moreover, the organization of standardized diabetes registers in an increasing number of countries can efficiently expand the approach to international comparisons of best practices.

### Methodological considerations

Concerning the publication bias funnel plots (Fig. 3), they showed small-study asymmetry on the left lower quadrant for the total LEA subgroup, accountable to two studies with lower quality scores. This suggests a potential publication bias for total LEA, where no small studies reporting negative results were found, and where small-study publications may potentially report overestimated results. On the contrary, major LEA remains unaffected by this publication bias. Sufficiently large sample sizes are required to report enough LEA cases to disaggregate results into major–minor LEA, and larger publications are likely to get published regardless of the magnitude of the outcome. This may explain why small observational studies were not found for this subgroup.

The percentage of agreement between authors was low-moderate (56%). This can be attributed to the variability in the definition of the interventions of interest in the individual studies, which required of several iterations between authors to reach a consensus.

### Limitations and strengths

Firstly, despite of the large number of studies identified, we were able to meta-analyze only on a limited number of studies. The studies selected may not be representative. To mitigate this, we described the composition of the entire set of papers, showing that the two groups were fairly comparable in terms of study design, characteristics of the organizational interventions, case-mix of subjects, state of managed care (primary vs secondary prevention), and target outcomes (minor and major LEA).

Secondly, the seven meta-analyzed studies did not differentiate minor and major LEA at a patient level, making it difficult to draw clinical conclusions on the potential effectiveness of selected care. We conclude future studies should analyze specific interventions at an individual patient level. This should consider the possibility of combinations of different amputations, which can be more easily identified in longitudinal cohort analyses based on EMRs extracted from routine databases.

Thirdly, the strength of any interpretation is limited by the heterogeneity found even among the seven meta-analyzed studies. We found a general lack of standardization in the definition and comparability of the interventions under study. However, with regards to the validity of the comparisons tested in the meta-analysis, at the level of each study, we may assume that there was a fair comparison between alternative care arrangements. This allows drawing valid conclusions considering health system features only, and not the broader determinants of disease [46] which we know are important [47].

Lastly, concerning publication bias, results for total LEA include small studies with inadequate sample sizes where not enough LEA cases were reported to disaggregate results in terms of minor–major LEA. However, results for the major LEA subgroup are of a larger positive magnitude and include only studies of adequate quality, with no suggestion of overestimated results or publication bias, bringing certainty to our conclusions.

## Conclusion

We identified that specific healthcare arrangements, which as main elements include care pathways, dedicated and multidisciplinary teams, and combined interventions, have the potential to cut the rate of major amputations in people with T2D and DFU in half.

Studies were heterogeneous in terms of design and definition of care arrangement, making it difficult to apply in context. Future research should focus on more comparable study designs, well-defined organizational targets, and a higher standardization or definition of care arrangements. This can be enabled through a better use of EMRs for cohort studies.

Reorganizing and optimising care arrangements and the use of a multidisciplinary approach are associated with improved outcomes for high-risk people with diabetes and foot ulceration. Future studies with more specific information on interventions are required to turn evidence into action, by tailoring care settings to the specific needs of patients that can benefit more.

## Supplementary information

Supplementary material 1 (DOC 102 kb)

Supplementary material 1 (DOCX 22 kb)

## Data Availability

All statistical analyses were performed using the statistical package R v.1.2 and the *metafor* package. All R code is available at request.
