# Supplementary material for: Health service organisation impact on lower extremity amputations in people with type 2 diabetes with foot ulcers: systematic review and meta-analysis
Source: Acta Diabetol. 2021 Feb 6;58(6):735–47. doi: 10.1007/s00592-020-01662-x (PMC7864802; doi:10.1007/s00592-020-01662-x)
Supplement: Supplementary file 1 — Supplementary material 1 (DOC 102 kb) [file 592_2020_1662_MOESM1_ESM.doc]

Data collection form for intervention reviews: RCTs and non-RCTs

This template was adapted for this specific systematic review and meta-analysis from the Cochrane collaboration’s “Data collection form for intervention reviews: RCTs and non-RCTs”, Version 3, April 2014.

| Review title or ID |  |
| --- | --- |
| Study ID *(surname of first author and year first full report of study was published e.g. Smith 2001)* |  |
| Notes | |

# General Information

| Date form completed *(dd/mm/yyyy)* |  |
| --- | --- |
| Name/ID of person extracting data |  |
| Publication type *(e.g. full report, abstract, letter)* |  |
| Notes: | |

# Study eligibility for qualitative analysis

| Study Characteristics | Eligibility criteria | | Eligibility criteria met? | | | Location in text or source |
| --- | --- | --- | --- | --- | --- | --- |
|  |  |  | Yes | No | Unclear |  |
| **Participants**: people with type two diabetes and presenting a foot ulcer as an index condition |  | |  |  |  |  |
| **Types of intervention**: any of four specific organizational arrangements (dedicated teams, multidisciplinary teams, care pathways, combined interventions) |  | |  |  |  |  |
| **Types of outcome measures**: Lower extremity amputation as primary endpoint). |  | |  |  |  |  |
| INCLUDE | | EXCLUDE | | | | |
| Reason for exclusion |  | | | | | |
| Notes: | | | | | | |

**DO NOT PROCEED IF STUDY EXCLUDED FROM REVIEW**

# Study eligibility for meta-analysis

| Study Characteristics | Report the number (e.g. n cases, n control) | | Eligibility criteria met? | | | Location in text or source |
| --- | --- | --- | --- | --- | --- | --- |
|  |  |  | Yes | No | Unclear |  |
| **Study presenting lower extremity amputation as primary endpoint in terms of quantitative measures** |  | |  |  |  |  |
| **Quantitative measures report the number of LEA cases as well as the number of persons at risk** (e.g. population as denominator, not as rate) |  | |  |  |  |  |
| **Types of comparison: the quantitative measures are reported for both an intervention and a control group** (either in parallel or before vs after) |  | |  |  |  |  |
| INCLUDE | | EXCLUDE | | | | |
| Reason for exclusion |  | | | | | |
| Notes: | | | | | | |

**PROCEED TO “OTHER INFORMATION” SECTION IF STUDY EXCLUDED FROM META-ANALYSIS**

# Characteristics of included studies

## Methods

|  | **Descriptions as stated in report/paper** | **Location in text or source** *(pg & ¶/fig/table/other)* |
| --- | --- | --- |
| **Aim of study** *(* |  |  |
| **Design** *(e.g. retrospective cohort, prospective observational, systematic review, audit, RCT)* |  |  |
| **Data collection methods** *(hospital records, EHRs, ICD-10 coding, survey)* |  |  |
| **Start date** |  |  |
| **End date** |  |  |
| **Duration of participation** *(from recruitment to last follow-up)* |  |  |
| **Comparison groups** *(single branch, parallel, before vs after)* |  |  |
| **Notes:** | | |

## Participants

|  | Description | Location in text or source |
| --- | --- | --- |
| Population description *(as described in study e.g. T2D, in-outpatient, active DFU)* |  |  |
| Country |  |  |
| Setting general *(primary-secondary care, community level)* |  |  |
| Setting specifics *(name and country/region of care unit)* |  |  |
| Inclusion criteria |  |  |
| Exclusion criteria |  |  |
| Method of inclusion of participants *(e.g. inpatient, emergency room for active ulcer)* |  |  |
| Age |  |  |
| Sex |  |  |
| Race/Ethnicity |  |  |
| Severity of illness / diabetic foot ulcer score reported? |  |  |
| Co-morbidities |  |  |
| Other relevant sociodemographics |  |  |
| Subgroups reported |  |  |
| Notes: | | |

## Intervention groups

**Intervention and control g**roup

|  | Description as stated in report/paper | Location in text or source |
| --- | --- | --- |
| Intervention description *(as described in study)* |  |  |
| Intervention category *(as to our categorization e.g. multidisciplinary teams)* |  |  |
| Is study comparing intervention group versus no intervention or standard care as a control group? (Y/N) |  |  |
| Control group description *(as described in study)* |  |  |
| Summary description of control group *(e.g. no intervention, best standard of care, routine care)* |  |  |
| Duration of intervention period |  |  |
| Timing *(e.g. frequency, duration of each episode)* |  |  |
| Delivery *(anything different from organized care intervention definitions used)* |  |  |
| Providers *(if different from intervention description)* |  |  |
| Notes: | | |

## Outcomes

**Outcome: Lower extremity amputation (LEA)**

|  | Description as stated in report/paper | Location in text or source |
| --- | --- | --- |
| LEA type reported (total, major, minor) |  |  |
| Time points reported |  |  |
| Outcome definition *(with diagnostic criteria if relevant)* |  |  |
| Unit of measurement *(OR, RR, N, LEA rate per 1000)* |  |  |
| Notes: | | |

#

# Other information

|  | **Description as stated in report/paper** | **Location in text or source** *(pg & ¶/fig/table/other)* |
| --- | --- | --- |
| **Key conclusions of study authors (as described in study)** |  |  |
| **Conclusion key point (as summarized by reviewer)** |  |  |
| **References to other relevant studies (snowballing)** |  |  |
| **Correspondence required for further study information** *(from whom, what and when)* |  | |
| **Notes:** | | |

#

**Sources:**

Cochrane Collaboration Glossary, 2010. Available from [www.cochrane.org/glossary](http://www.cochrane.org/glossary).

Higgins JPT, Green S (editors). Cochrane Handbook for Systematic Reviews of Interventions Version 5.1.0 [updated March 2011]. The Cochrane Collaboration, 2011. Available from [handbook.cochrane.org](http://handbook.cochrane.org/).

Last JM (editor), A Dictionary of Epidemiology, 4^th^ Ed. New York: Oxford University Press, 2001.

Schünemann H, Brożek J, Oxman A, editors. GRADE handbook for grading quality of evidence and strength of recommendation. Version 3.2 [updated March 2009]. The GRADE Working Group, 2009.
