# Supplementary material for: Health service organisation impact on lower extremity amputations in people with type 2 diabetes with foot ulcers: systematic review and meta-analysis
Source: Acta Diabetol. 2021 Feb 6;58(6):735–47. doi: 10.1007/s00592-020-01662-x (PMC7864802; doi:10.1007/s00592-020-01662-x)
Supplement: Supplementary file 2 — Supplementary material 1 (DOCX 22 kb) [file 592_2020_1662_MOESM2_ESM.docx]

| Title | Reference | Authors | Evidence | Intervention |
| --- | --- | --- | --- | --- |
| Negative pressure wound therapy for treating foot wounds in people with diabetes mellitus. | Cochrane Database Syst Rev. 2018 Oct 17;10:CD010318. | Liu Z, Dumville JC, Hinchliffe RJ, Cullum N, Game F, Stubbs N, Sweeting M, Peinemann F. | 2. Weak against | combined intervention |
| Clinical outcomes among morbidly obese patients hospitalized with diabetic foot complications. | Clin Obes. 2019 Feb;9(1):e12285. | Harris CM, Abougergi MS, Wright S. | 4. Weak support | care pathways |
| Diabetic foot management: multidisciplinary approach for advanced lesion rescue. | J Cardiovasc Surg (Torino). 2018 Oct;59(5):670-684. | Brocco E, Ninkovic S, Marin M, Whisstock C, Bruseghin M, Boschetti G, Viti R, Forlini W, Volpe A. | 4. Weak support | multidisciplinary teams |
| Reducing Amputations in People with Diabetes (RAPID): Evaluation of a New Care Pathway. | Int J Environ Res Public Health. 2018 May 16;15(5). pii: E999. | MacRury S, Stephen K, Main F, Gorman J, Jones S, Macfarlane D. | 4. Weak support | care pathways |
| Barriers to foot care in patients with diabetes as identified by healthcare professionals. | Diabet Med. 2018 Aug;35(8):1072-1077. | Pankhurst CJW, Edmonds ME. | 4. Weak support | care pathways |
| The Society for Vascular Surgery Wound, Ischemia, and foot Infection (WIfI) classification system correlates with cost of care for diabetic foot ulcers treated in a multidisciplinary setting. | J Vasc Surg. 2018 May;67(5):1455-1462. | Hicks CW, Canner JK, Karagozlu H, Mathioudakis N, Sherman RL, Black JH 3rd, Abularrage CJ. | 5. Strong support | care pathways |
| Diabetes-related major lower limb amputation incidence is strongly related to diabetic foot service provision and improves with enhancement of services: peer review of the South-West of England. | Diabet Med. 2018 Jan;35(1):53-62. Erratum in: Diabet Med. 2018 Mar;35(3):394. | Paisey RB, Abbott A, Levenson R, Harrington A, Browne D, Moore J, Bamford M, Roe M; South-West Cardiovascular Strategic Clinical Network peer diabetic foot service review team. | 5. Strong support | care pathways |
| Intensive versus conventional glycaemic control for treating diabetic foot ulcers. | Cochrane Database Syst Rev. 2016 Jan 13;(1):CD010764. | Fernando ME, Seneviratne RM, Tan YM, Lazzarini PA, Sangla KS, Cunningham M, Buttner PG, Golledge J. | 3. Inconclusive evidence | combined intervention |
| Diabetic foot infections: what have we learned in the last 30 years? | Int J Infect Dis. 2015 Nov;40:81-91. | UÃ§kay I, AragÃ³n-SÃ¡nchez J, Lew D, Lipsky BA. | 3. Inconclusive evidence | combined intervention |
| When and how to audit a diabetic foot service. | Diabetes Metab Res Rev. 2016 Jan;32 Suppl 1:311-7. | Leese GP, Stang D. | 4. Weak support | care pathways |
| Gender differences in the relationship between diabetes process of care indicators and cardiovascular outcomes. | Eur J Public Health. 2016 Apr;26(2):219-24. | Seghieri C, Policardo L, Francesconi P, Seghieri G. | 4. Weak support | combined intervention |
| Complex interventions for preventing diabetic foot ulceration. | Cochrane Database Syst Rev. 2015 Aug 24;(8):CD007610. | Hoogeveen RC, Dorresteijn JA, Kriegsman DM, Valk GD. | 3. Inconclusive evidence | combined intervention |
| Amputation Risk in Patients with Diabetes Mellitus and Peripheral Artery Disease Using Statewide Data. | Ann Vasc Surg. 2016 Jan;30:123-31. | Humphries MD, Brunson A, Hedayati N, Romano P, Melnkow J. | 1. Strong against | combined intervention |
| Organization of wound healing services: The impact on lowering the diabetes foot amputation rate in a ten-year review and the importance of early debridement. | Diabetes Res Clin Pract. 2015 Jul;109(1):77-84. | Hsu CR, Chang CC, Chen YT, Lin WN, Chen MY. | 5. Strong support | multidisciplinary teams |
| Limited evidence to assess the impact of primary health care system or service level attributes on health outcomes of Indigenous people with type 2 diabetes: a systematic review. | BMC Health Serv Res. 2015 Apr 11;15:154. | Gibson OR, Segal L. | 3. Inconclusive evidence | combined intervention |
| Non-healing foot ulcers in diabetic patients: general and local interfering conditions and management options with advanced wound dressings. | J Wound Care. 2015 Apr;24(4 Suppl):35-42. | Uccioli L, Izzo V, Meloni M, Vainieri E, Ruotolo V, Giurato L. | 4. Weak support | combined intervention |
| Reduction in diabetic amputations over 15 years in a defined Spain population. Benefits of a critical pathway approach and multidisciplinary team work. | Rev Esp Quimioter. 2014 Sep;27(3):170-9. | MartÃ­nez-GÃ³mez DA, Moreno-Carrillo MA, Campillo-Soto A, Carrillo-GarcÃ­a A, Aguayo-Albasini JL. | 5. Strong support | combined intervention |
| Primary care diabetes bundle management: 3-year outcomes for microvascular and macrovascular events. | Am J Manag Care. 2014 Jun 1;20(6):e175-82. | Bloom FJ Jr, Yan X, Stewart WF, Graf TR, Anderer T, Davis DE, Pierdon SB, Pitcavage J, Steele GD Jr. | 5. Strong support | care pathways |
| Diabetic foot ulcers: evaluation and management. | Hosp Pract (1995). 2012 Aug;40(3):102-15. | Maderal AD, Vivas AC, Zwick TG, Kirsner RS. | 4. Weak support | combined intervention |
| 2012 Infectious Diseases Society of America clinical practice guideline for the diagnosis and treatment of diabetic foot infections. | Clin Infect Dis. 2012 Jun;54(12):e132-73. | Lipsky BA, Berendt AR, Cornia PB, Pile JC, Peters EJ, Armstrong DG, Deery HG, Embil JM, Joseph WS, Karchmer AW, Pinzur MS, Senneville E; Infectious Diseases Society of America.. | 5. Strong support | multidisciplinary teams |
| The impact of adherence to screening guidelines and of diabetes clinics referral on morbidity and mortality in diabetes. | PLoS One. 2012;7(4):e33839. | Giorda C, Picariello R, Nada E, Tartaglino B, Marafetti L, Costa G, Gnavi R. | 5. Strong support | care pathways |
| A diabetic foot service established by a department of vascular surgery: an observational study. | Ann Vasc Surg. 2012 Jul;26(5):700-6. | Williams DT, Majeed MU, Shingler G, Akbar MJ, Adamson DG, Whitaker CJ. | 5. Strong support | multidisciplinary teams |
| The impact and outcomes of establishing an integrated interdisciplinary surgical team to care for the diabetic foot. | Diabetes Metab Res Rev. 2012 Sep;28(6):514-8. | Armstrong DG, Bharara M, White M, Lepow B, Bhatnagar S, Fisher T, Kimbriel HR, Walters J, Goshima KR, Hughes J, Mills JL. | 5. Strong support | multidisciplinary teams |
| Wound healing--a practical algorithm. | Diabetes Metab Res Rev. 2012 Feb;28 Suppl 1:85-8. | Jeffcoate WJ. | 4. Weak support | multidisciplinary teams |
| Cost-effectiveness of a shared computerized decision support system for diabetes linked to electronic medical records. | J Am Med Inform Assoc. 2012 May-Jun;19(3):341-5. | O'Reilly D, Holbrook A, Blackhouse G, Troyan S, Goeree R. | 4. Weak support | care pathways |
| The diabetic foot: an overview of assessment and complications. | Br J Nurs. 2011 Aug 11-Sep 8;20(15):S19-25. | Turns M. | 4. Weak support | combined intervention |
| Diagnostics, theragnostics, and the personal health server: fundamental milestones in technology with revolutionary changes in diabetic foot and wound care to come. | Foot Ankle Spec. 2011 Feb;4(1):54-60. | Armstrong DG, Giovinco NA. | 4. Weak support | combined intervention |
| Improving the efficiency and effectiveness of performing the diabetic foot exam. | Am J Med Qual. 2011 May-Jun;26(3):193-9. | Praxel TA, Ford TJ, Vanderboom EW. | 4. Weak support | care pathways |
| A step-wise approach for surgical management of diabetic foot infections. | J Vasc Surg. 2010 Sep;52(3 Suppl):72S-75S. | Fisher TK, Scimeca CL, Bharara M, Mills JL Sr, Armstrong DG. | 4. Weak support | multidisciplinary teams |
| The costs of diabetic foot: the economic case for the limb salvage team. | J Vasc Surg. 2010 Sep;52(3 Suppl):17S-22S. | Driver VR, Fabbi M, Lavery LA, Gibbons G. | 5. Strong support | care pathways |
| Diabetes outpatient care before and after admission for diabetic foot complications. | J Wound Care. 2010 Apr;19(4):150-2. | Ellis E, Ballance K, Lunt H, Lewis D. | 4. Weak support | combined intervention |
| Foot ulcers in the diabetic patient, prevention and treatment. | Vasc Health Risk Manag. 2007;3(1):65-76. | Wu SC, Driver VR, Wrobel JS, Armstrong DG. | 4. Weak support | combined intervention |
| Factors Associated With Ulcer Healing and Quality of Life in Patients With Diabetic Foot Ulcer. | Angiology. 2017 Mar;68(3):242-250. | Spanos K, Saleptsis V, Athanasoulas A, Karathanos C, Bargiota A, Chan P, Giannoukas AD. | 4. Weak support | dedicated teams |
| An integrated care pathway to save the critically ischaemic diabetic foot. | Int J Clin Pract. 2006 Jun;60(6):667-9. | El Sakka K, Fassiadis N, Gambhir RP, Halawa M, Zayed H, Doxford M, Greensitt C, Edmonds M, Rashid H. | 5. Strong support | multidisciplinary teams |
| Evidence-based protocol for diabetic foot ulcers. | Plast Reconstr Surg. 2006 Jun;117(7 Suppl):193S-209S; discussion 210S-211S. Review. | Brem H, Sheehan P, Rosenberg HJ, Schneider JS, Boulton AJ. | 4. Weak support | care pathways |
| Gender differences across racial and ethnic groups in the quality of care for diabetes. | Womens Health Issues. 2006 Mar-Apr;16(2):56-65. | Correa-de-Araujo R, McDermott K, Moy E. | 4. Weak support | combined intervention |
| The relationship between provider coordination and diabetes-related foot outcomes. | Diabetes Care. 2003 Nov;26(11):3042-7. | Wrobel JS, Charns MP, Diehr P, Robbins JM, Reiber GE, Bonacker KM, Haas LB, Pogach L. | 5. Strong support | care pathways |
| What is the most effective way to reduce incidence of amputation in the diabetic foot? | Diabetes Metab Res Rev. 2000 Sep-Oct;16 Suppl 1:S75-83. Review. | Apelqvist J, Larsson J. | 4. Weak support | multidisciplinary teams |
| Evaluation of the effect of performance monitoring and feedback on care process, utilization, and outcome. | Diabetes Care. 2000 Feb;23(2):192-6. | Petitti DB, Contreras R, Ziel FH, Dudl J, Domurat ES, Hyatt JA. | 3. Inconclusive evidence | combined intervention |
| Critical pathway approach to diabetic pedal infections in a multidisciplinary setting. | J Foot Ankle Surg. 1999 Jan-Feb;38(1):30-3; discussion 82-3. | Crane M, Werber B. | 5. Strong support | care pathways |
| Impact of multidisciplinary foot care team on outcome of diabetic foot ulcer in term of lower extremity amputation at a tertiary care unit in Karachi, Pakistan | International Wound Journal (2019) 16:3 (768-772). | Riaz M., Miyan Z., Waris N., Zaidi S.I.H., Tahir B., Fawwad A., Basit A. | 5. Strong support | combined intervention |
| Pathway to ending avoidable diabetes-related amputations in Australia | The Medical journal of Australia (2018) 209:7 (288-290). | Lazzarini P.A., van Netten J.J., Fitridge R.A., Griffiths I., Kinnear E.M., Malone M., Perrin B.M., Prentice J., Wraight P.R. | 2. Weak against | care pathways |
| Fighting diabetic foot ulcers—The diabetologist: A king maker of the fight | Seminars in Vascular Surgery (2018) 31:2-4 (49-55). | Nigi L., Fondelli C., de Donato G., Palasciano G., Setacci C., Dotta F. | 4. Weak support | multidisciplinary teams |
| Current challenges and opportunities in the prevention and management of diabetic foot ulcers | Diabetes Care (2018) 41:4 (645-652). | Jeffcoate W.J., Vileikyte L., Boyko E.J., Armstrong D.G., Boulton A.J.M. | 4. Weak support | care pathways |
| Reducing major lower extremity amputations after the introduction of a multidisciplinary team in patient with diabetes foot ulcer | BMC Endocrine Disorders (2016) 16:1 Article Number: 38. | Wang C., Mai L., Yang C., Liu D., Sun K., Song W., Luo B., Li Y., Xu M., Zhang S., Li F., Ren M., Yan L. | 5. Strong support | multidisciplinary teams |
| Reducing amputations in the UK: A key to unlock the door | Diabetic Medicine (2015) 32 SUPPL. 1 (154). | Gandhi R.A., Selvarajah D., McDonnell R.A., Cerrone E., Creagh F.M. | 2. Weak against | care pathways |
| Improving a diabetes foot service to prevent amputations: The Portsmouth experience | British Journal of Diabetes and Vascular Disease (2015) 15:4 (180-183). | Humayun M.A., Meeking D. | 5. Strong support | care pathways |
| The pathway to amputation in patients with diabetes; missed opportunities for early intervention and implications on clinical management | Diabetic Medicine (2014) 31 SUPPL. 1 (124). | Ali M.A., Higgins K.S., Webb D.R., Davies M.J., Choke E., Sayers R.D., Bown M.J. | 4. Weak support | care pathways |
| Preventing the first or recurrent ulcers | Medical Clinics of North America (2013) 97:5 (808-820). | Lavery L.A., La Fontaine J., Kim P.J. | 4. Weak support | combined intervention |
| Value of team approach combined with clinical pathway for diabetic foot problems: A clinical evaluation | Diabetic Foot and Ankle (2010) 1. Date of Publication: 2010 | Nather A., Bee C.S., Lin W.K., Valerie C.X.-B., Liang S., Tambyah P.A., Jorgensen A., Nambiar A. | 5. Strong support | combined intervention |
| Assessing the outcome of the management of diabetic foot ulcers using ulcer-related and person-related measures | Diabetes Care (2006) 29:8 (1784-1787). Date of Publication: 2006 | Jeffcoate W.J., Chipchase S.Y., Ince P., Game F.L. | 4. Weak support | combined intervention |
| Development of a clinical pathway for diabetic foot | Revista de Calidad Asistencial (2003) 18:4 (235-243). | De Alcalá D., Aguayo J.L., Soria V., Illán F., Aguirán L.M., Pérez-Abad J.M., Andreo J.A. | 4. Weak support | multidisciplinary teams |
| Two-Year Foot Care Program for Minority Patients with Type 2 Diabetes Mellitus of Zhuang Tribe in Guangxi, China. | Can. J. Diabetes 36, 15–18 (2012). | Liang, R., Dai, X., Zuojie, L., Zhou, A. & Meijuan, C. | 5. Strong support | multidisciplinary teams |
| Diabetes education and care management significantly improve patient outcomes in the dialysis unit. | Am. J. Kidney Dis. 40, 566–575 (2002) | McMurray, S. D., Johnson, G., Davis, S. & McDougall, K. | 5. Strong support | combined intervention |
| Improvement of diabetic foot care after the Implementation of the International Consensus on the Diabetic Foot (ICDF): Results of a 5-year prospective study. | Diabetes Res. Clin. Pract. 75, 153–158 (2007). | Anichini, R. et al. | 5. Strong support | multidisciplinary teams |
| Reduction in diabetic amputations over 11 years in a defined U.K. population: benefits of multidisciplinary team work and continuous prospective audit. | Diabetes Care 31, 99–101 (2008). | Krishnan, S., Nash, F., Baker, N., Fowler, D. & Rayman, G. | 5. Strong support | multidisciplinary teams |
| Disease management for the diabetic foot: effectiveness of a diabetic foot prevention program to reduce amputations and hospitalizations | Diabetes Research and Clinical Practice, 31 Mar 2005, 70(1):31-37 | Lavery LA, Wunderlich RP, Tredwell JL | 5. Strong support | care pathways |
